# Effective Response to Hospital Congestion Scenarios: Simulation-Based Evaluation of Decongestion Interventions

**DOI:** 10.3390/ijerph192316348

**Published:** 2022-12-06

**Authors:** Wanxin Hou, Shaowen Qin, Campbell Henry Thompson

**Affiliations:** 1 School of Information Science and Technology, Research Centre for Intelligent Information Technology, Nantong University, Nantong 226019, China; 2 College of Science and Engineering, Flinders University, Adelaide 5042, Australia; 3 School of Medicine, University of Adelaide, Adelaide 5005, Australia

**Keywords:** hospital congestion, simulation model, decongestion scenarios, efficiency

## Abstract

Hospital overcrowding is becoming a major concern in the modern era due to the increasing demand for hospital services. This study seeks to identify effective and efficient ways to resolve the serious problem of congestion in hospitals by testing a range of decongestion strategies with simulated scenarios. In order to determine more efficient solutions, interventions with smaller changes were consistently tested at the beginning through a simulation platform. In addition, the implementation patterns were investigated, which are important to hospital managers with respect to the decisions made to control hospital congestion. The results indicated that diverting a small number of ambulances seems to be more effective and efficient in congestion reduction compared to other approaches. Furthermore, instead of implementing an isolated approach continuously, combining one approach with other strategies is recommended as a method for dealing with hospital overcrowding.

## 1. Introduction

Hospitals are facing the challenge of an ever-increasing demand for healthcare services. This is compounded by inadequate resources and hospital overcrowding, which result in delayed treatment, an increased number of hospital infections, and patient mortality [1,2]. Therefore, the exploration of efficient approaches to tackling this negative issue is urgent for healthcare administrators.

Some researchers have attempted to identify the reasons for hospital congestion and to improve the efficiency of hospital operations using analytical approaches. Two examples of these approaches are regression modelling and time-series modelling [3,4]. Undoubtedly, it is quite challenging for researchers to model a hospital only through analytical methods due to a hospital’s structural and behavioral complexities [5]. In addition, numerical analysis-based methods often involve complicated concepts and algorithms that are not interpretable by healthcare professionals. Thus, simulation modeling is frequently chosen to test the effectiveness of solutions for congested flow in hospitals due to its unparalleled advantages in representing structurally complicated systems with a dynamically probabilistic and uncertain nature [6]. The properties of all the model components are stochastically assigned and updated according to the simulated environment over time [7]. It also allows for “strategical interaction”, which is important when a comparison between different strategies is required [8]. However, improving the accuracy of simulation-based evaluation is still a challenge faced by researchers. Among the responses to access block in hospitals, most studies have only focused on exploring solutions through simulation modelling of the Emergency Department (ED) [9,10,11,12,13], while interactions between the ED and other departments are often ignored. As a result, the precision of decongestion strategies based on the simulation model will be negatively impacted. To identify a more reliable end-to-end solution, the construction of a simulation model of the whole hospital is suggested [8]. The other issue regarding a simulation model’s construction is improving the model’s reliability. Due to a lack of necessary data, experts’ estimations and assumptions were often relied on for setting the parameters of a given simulation model. In addition, patients’ behaviors or treatment processes were simulated based on a fixed pattern, which lowers a model’s reliability [14]. Therefore, modelling the trajectories of individual behavioral and process-related change over time more realistically and comprehensively is the crucial direction of effort in this field.

Different strategies have been proposed for reducing hospital congestion, including resource optimization, additional resources, patient discharging, and process improvement [15,16]. Researchers normally apply analytical or statistical methods to generate the optimal pattern for resources’ reallocation or process improvement and then test the effectiveness of these approaches through a simulation model [17,18]. Undoubtedly, from the results’ perspective, selecting an approach that can reduce congestion as much as possible is the goal of hospital stockholders. However, from a practical perspective, these approaches might lead to massive staff and structural changes that lead to certain difficulties with respect to their realization or generate the potential risk of financial burden. Therefore, decongestion interventions that cause fewer disruptions (i.e., implementing smaller changes) while achieving similar outcomes are more desirable. As a result, exploring more efficient interventions is the focus of this study.

The isolated interventions dealing with hospital congestion issues have been estimated and compared [15,19,20]. Without doubt, hospital mangers can continuously carry out the same approach. They only need to increase the intensity of the approach execution, such as discharging more patients or adding more beds if the situation requires more interventions to control. The other choice for managers is combining different approaches together to deal with the congestion issue. When mangers attempt to implement interventions for hospital overcrowding control, an issue might emerge regarding the efficiency of the two implementation schemes and which one should be focused on. Consequently, this study aimed to explore more efficient and effective decongestion interventions based on a simulation model and offer objective reasoning to support hospital management decisions. In this study, a more realistic simulation model for a whole hospital that captures the patterns of patients’ flow based on real time-series data [21] was used to evaluate decongestion strategies. All the interventions were tested through smaller adjustments to avoid wasting resources. Indicators were selected from different hospital units to measure congestion statuses and to compare the efficiency of different decongestion strategies activated soon after congestions occur in the hospitals. Furthermore, the efficiencies of different strategy evaluation patterns were also discussed. The rest of this paper is organized as follows: Section 2 mainly introduces the simulation model and the decongestion strategies. Section 3 describes the simulation results so that the solutions can be compared and considered for real-world implementation. Finally, the discussion and conclusions are presented in Section 4 and Section 5.

## 2. Materials and Methods

### 2.1. Hospital Simulation Model

**Figure 1:**
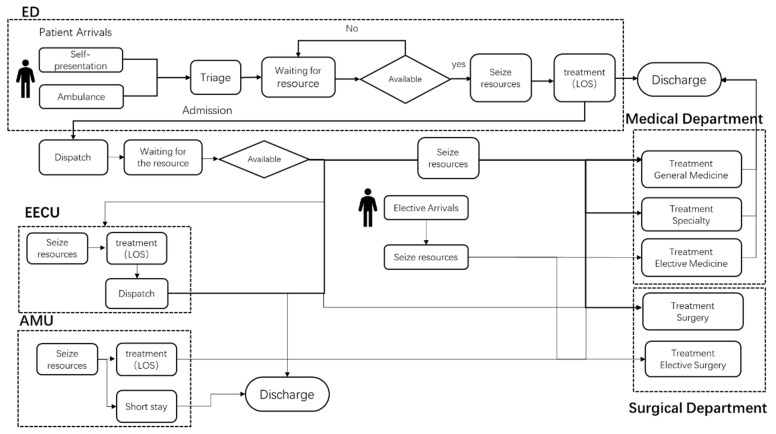
Patient flow of the system.

Simulation modeling provides an opportunity to examine the behavior of complex systems as a whole and offers a risk-free platform to evaluate interventions in a hospital. In this study, a simulation model has been developed to imitate the patterns of patient flow through a large tertiary hospital in Australia based on 2-year patient journey data (2014–2016) [21]. It adopted a modular structure (a module represents either a physical unit or a process) and captures different types of patients’ pathways through the hospital from the time they first arrived in the ED, or were admitted as elective patients, to the time that they were discharged. The patient flow of the system is shown in Figure 1. Patient “agents” are assigned their own properties, which are used for tracking milestones in the patient’s journey. These milestones include which units of the hospital they enter and at what times they complete various aspects of their journey (more information is described in Supplementary Information SI1 and SI2). The physical units of the hospital are described in Table S1 (Supporting Information). The model allows for a realistic representation of patient flows at a level of resolution that was deemed appropriate by the hospitals’ data management experts, balancing complexity and fidelity. It has been validated against historical data and through consultation with health care and hospital experts [21].

### 2.2. Decongestion Evaluations

The simulation model provides a risk-free platform from which to investigate decongestion strategies when congestion occurs in a hospital. Initially, it was essential for this study to define the criteria for what is considered hospital congestion. In most cases, smoothing patient flow in hospitals equates to minimizing ED patients’ queue length, length of stay, and average waiting time [3]. For the whole hospital, it is necessary to select indicators not only from ED but also from other departments to represent the hospital’s congestion status. In this study, three indicators were chosen to define hospital congestion: occupancy level of inpatient beds, utilization of ED treatment space, and the queue length of ED patients waiting for treatment. The congestion status was identified as the state wherein almost all beds (≥17 beds) in the ED’s admission side are occupied (18 beds in total), and wherein the bed occupancy in the inpatient department is greater than or equal to 0.97. In addition, a so-called reverse-engineering approach was applied to determine the ED queue length. We assumed that the frequency of congestion without implementing any decongestion interventions is approximately once a week, and based on the setting of the very high ED and hospital occupancy level, an average queue length can be derived for congestion of such severity. Finally, an average queue length of 28 was calculated. Without doubt, the definition of a congestion will always be subjective. It is the consistency that matters when the purpose is to compare the effects of different decongestion strategies on a relative scale. For example, we can set the queue length criterion to a smaller number, with overcrowding events occurring much frequently than once a week during the simulation period, and still compare the relative effectiveness of the decongestion strategies.

The intervention schemes for congestion prevention suggested by senior hospital staff were transcribed into scenarios for investigation in the simulation model. Some of them, such as moving ED patients or postponing elective patients, have been implemented in the hospital, while others are planned to be implemented. Table 1 shows all decongestion scenarios executed in this study’s simulation model including right sizing ED and inpatient wards, reducing admission (ED out-transfers and ambulance diversion), promoting discharges, and postponing elective admissions. We compared the degree (2, 4, and 6) of interventions of each type due to the fact that smaller adjustments were always promoted at the beginning, which are easier to implement. In addition, investigating smaller adjustments is the first and foremost method with which to avoid wasting resources. In some cases, analyzing more patients and physical resources might improve outcomes but sometimes this might lower efficiency.

Hospital capacity is an important factor that influences hospital overcrowding (hospital capacity: Table S1). Right sizing ED or inpatient departments strategies were tested in the model (scenario 1–12). In this study, 2, 4, and 6 beds were added to ED and AMU (Scenario 1–6). For inpatient departments, the total numbers of beds are unchanged; we slightly adjusted the number of surgical and medical beds (Scenario 7–12).

Moreover, hospital staff control congestion through adjustment of patients. Scenarios 13–15 explored moving ED patients to other hospitals. Scenarios 16–18 discharge 2, 4, and 6 inpatients when congestion appears. Scenarios 19 to 21 tested the effects of deferring the admission of elective patients when congestions occur. The model postponed 2, 4, and 6 surgical elective patients during a congestion episode. Instead of removing these patients, the model placed them in a backlog of elective patients waiting for rescheduling when beds became available in the hospital. Scenarios 22–23 investigated the impact of diverting ambulance arrivals during a congestion episode. In a real situation, hospital staff will not discharge or postpone patients with severe illnesses. To realize a process as realistic as possible in the simulation model, patients that arrived to the hospital were assigned a triage score (from 1 to 5), which is used as an indicator of the severity of a patient’s condition. A low score indicates that patients require immediate treatment, and a high score corresponds to a less urgent case. The model firstly selected patients whose triage score was greater than or equal to 4 for discharging. Scenarios 24–25 are different combinations of decongestion approaches.

The simulation model checked the congestion status each hour. The interventions were activated one hour after congestion occurred. Each scenario simulated two years of hospital operations. However, only the results of the second year were collected to minimize the impact of the ‘warm-up’ period. In addition, each scenario was replicated 20 times under the same conditions to obtain an average behavior that would allow for meaningful comparison of the results from different intervention scenarios.

The effect of each scenario on decongestion was mainly estimated through the number of congestion events over a one-year period. Congestion time is the other indicator, which represents the duration of congestion. Reducing congestion time allows the hospital to operate at its designed capacity and maintain the quality of its service. Other measures reported were 10 am hospital occupancy, affected patients, postponed elective patients, and diverted ambulance arrivals. All of them are important measures for gaining insight into the potential impacts of each decongestion strategy. To provide more objective comparison of the scenarios, the reduction in congestion numbers or congestion time per person affected in each scenario were evaluated. The most beneficial intervention is the one that not only improves reduction outcomes (more effective) but also affects fewer patients (more efficient), in other words, when the congestion reduction per affected patient is higher compared to other interventions.

## 3. Results

The simulation results of the different decongestion scenarios are shown in Table 1. The change of hospital occupancy is not obvious while implementing those decongestion interventions. Those interventions that involved ambulance diversion were most effective at reducing the annual frequency of congestion episodes, especially those interventions that combined ambulance diversions with other strategies such as ED out-transfers. Slightly less effective at reducing congestion frequency was the opening of new AMU beds followed by the slightly less effective strategy of opening new ED beds. The next most effective intervention for reducing congestion frequency was the transfer of admitted ED patients to another hospital. The postponement of elective surgeries and the earlier discharge of admitted inpatients were the next most helpful strategies for lowering the frequency of hospital congestions below the baseline. Re-sizing medical and surgical units barely changed the frequency of hospital congestion episodes from the baseline levels; perhaps increasing medical beds at the expense of surgical ones was more effective than vice versa.

Again, any intervention involving an ambulance diversion was the most effective at reducing the maximum duration of a congestion episode; perhaps ambulance diversion combined with another intervention was not especially better than the ambulance diversion on its own. Elective surgery postponement followed by opening AMU beds were the next most effective strategies for shortening maximum congestion duration and none of the remaining strategies significantly altered the maximum congestion duration below the pre-existing levels.

Finally, when assessing the relative effectiveness of the various interventions on the mean durations of congestion episodes, we developed a ranking scheme, which was very close to that seen above for the effectiveness of these strategies towards the maximum duration of congestion episodes.

Scenarios 13–26 were the only interventions that involved direct and significant changes to patient care. Hence, these were the only scenarios where the efficiency of each intervention (efficiency expressed as a function of the number of patients affected) could be gauged and compared.

**Figure 2:**
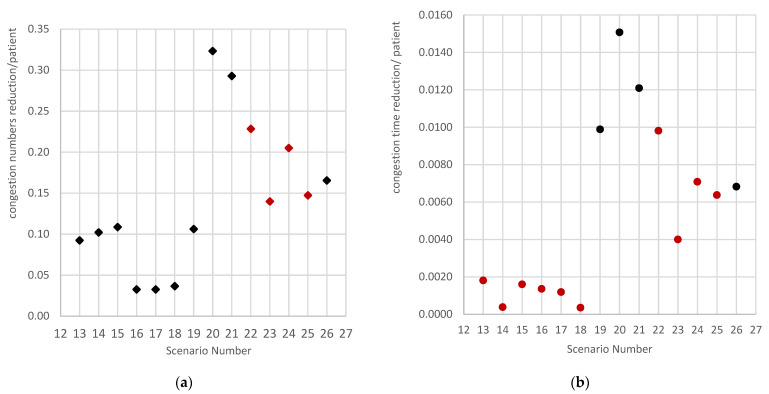
Efficiency of different scenarios. (**a**) Congestion numbers. (**b**) Mean congestion time.

The efficiencies of some decongestion interventions were also described in Figure 2. Efficiency was defined as the reduction in congestion per affected patient. Figure 2 indicates that postponing four elective surgical patients (Scenario 20) is the most effective way to reduce congestion frequency. The red points in Figure 2 mean the efficiency is decreasing if more patients are involved. Regarding the inventions concerning the diversion of ambulances, a smaller adjustment (diverting two ambulances) is more effective at decreasing the number of congestion events. According to Figure 2b, the combination of moving two ED patients and diverting two ambulances (scenario 25) reduces congestion time most effectively. Furthermore, discharging smaller numbers of ED patients and inpatients and diverting smaller numbers of ambulances would yield more efficient reductions in congestion duration (see Figure 2b).

## 4. Discussion

In this study, in order to deal with an increasing number of congestion issues, an integrated simulation model for a whole hospital that captures the patterns of patient flows based on real time-series data was applied to evaluate decongestion strategies. The findings indicated that all decongestion interventions except surgical bed expenses can reduce the annual congestion frequency and mean congestion duration, although the efficiency of each of these scenarios is distinct. Surprisingly, some of the decongestion strategies boost the maximum congestion time duration, such as moving a small number of ED patients and inpatients when congestion occurs. Such non-intuitive results discovered through the simulation-based evaluation indicated that moving ED patients is a preferable method for controlling congestion from a management viewpoint; however, these strategies might lead to patients’ dissatisfaction due to the likely longer waiting times.

This study estimated different methods for tackling hospital overcrowding by adding small quantities of ED beds and AMU beds, removing ED patients, discharging inpatients, postponing elective patients, diverting ambulances, or combinations of them. The implementation of a single strategy, namely, adding AMU beds, seems to be the best way to reduce hospital congestion. It can be seen that the medical-related units might have an appreciable impact on hospital overcrowding due to the fact that the AMU is an assessment unit for general medical and acute care elderly patients. The study by Hammer et al. also pointed out that increasing the number of inpatient beds helps decrease hospital overcrowding, especially in the ED access block [22]. Further evidence was provided by moving surgical beds to the medical department (scenarios 7–9). The simulation-based evaluation pointed out that right-sizing the medical department brings more expected benefits with respect to congestion reduction compared to other inpatient departments. By tracing the historical data, it has been found that there are 17.3% more medical patients than surgical patients (Table S3).

By comparing the relocation of ED patients to the addition of ED beds, we discovered that adjusting the size of physical units was more effective for congestion reduction than for operating patients. We considered that adding a bed might benefit a considerable number of patients during a period of hospital congestion. Moving or discharging patients seems to have fewer effects because it only involves certain numbers of discharged patients. However, the studies by Salway et al. concluded that increasing the number of ED beds might increase the number of boarders [23,24]. Therefore, they pointed out that adding beds to the ED is not a satisfactory solution because they only applied the number of boarders to evaluate overcrowding. In our study, hospital congestion was defined as not only the occupancies but also the patient queue length while waiting for treatment. In some cases, increasing the number of ED beds might not decrease the occupancy, but it can decrease the queue length according to our results. Without a doubt, it is important to recognize that opening a bed requires more costs. Hence, from a cost–benefit perspective, the profits and costs from the decongestion effects of the scenarios need to be explored further in future tasks.

In a real situation, decongestion strategies are often implemented according to two patterns. One is continuously carrying out the same approach for a specific department. Hospital managers only need to increase the intensity of the approach’s execution, such as discharging more patients or adding more beds for specific departments if the situation requires more interventions to control. The other pattern is combining different approaches together to deal with the congestion issue. In this study, our estimation indicated that diverting ambulances can greatly decrease congestion frequency as well as congestion duration. Acuna et al. also evidenced that the optimal allocation of ambulances can markedly reduce the waiting time for treatment and decrease overcrowding in the healthcare system [25,26]. Diverting smaller numbers of ambulances (e.g., diverting two arrivals) seems to be more efficient for the reduction of both congestion time and duration compared to combination scenario 24 (the efficiency of scenario 22 > 24). However, when we were continually diverting ambulances (e.g., diverting four arrivals), the efficiency decreased slightly. Therefore, if the congestion situation requires further interventions to control, instead of implementing an isolated approach continuously, combining one approach with other strategies is preferable because its efficiency is higher (the efficiency of scenario 26 > 25 > 23), as shown in Table 2.

An interesting discovery from this study is that the strategies that cause large adjustments can effectively reduce congestion episodes; however, they might not the most efficient ones. For some strategies, such as ambulance diversion and its combination with moving ED patients, a smaller change is often enough, and more cost effective, with respect to reducing hospital congestion issues (Figure 2). Moving smaller numbers of ED patients or inpatients are also more efficient strategies compared with further discharges.

The change in hospital occupancy is not obvious while implementing these decongestion interventions. However, it is different from existing studies showing that discharging patients can remarkably lower hospital capacity [21,27]. The reason for the difference is that the discharging scenarios were designed without considering the discharge condition in their studies. Therefore, the discharge of patients occurred throughout the whole simulation period. In addition, the patients might have been discharged when they were admitted immediately. Thus, the midnight occupancy drops significantly, which is different from this study. In this study, hospital congestion was defined by three indicators that provided the condition to execute interventions.

From a managerial perspective, we suggest that right sizing ED and inpatient department is very effective for controlling hospital overcrowding. In addition, hospital managers might need to pay more attention to the elderly, who might have certain contributions to hospital overcrowding. Without a doubt, right sizing hospital physical units is an efficient way to deal with congestion issues. However, opening a bed for patients requires greater costs. On the other hand, more efficient decongestion strategies and smoother patient flow may bring more benefits for the hospital. Therefore, from a profit perspective, managers are recommended to perform further evaluations before adding beds to hospital’s physical units. Finally, we suggest that managers combine different strategies rather than a single strategy to solve congestion problems. This is because combining different interventions may lead to more efficient outcomes.

This study has potential limitations. Without a doubt, hospital staff are very important hospital resources that play an important role in smoothing patient flow. However, in our hospital simulation model, we only simulated hospital beds for resource allocation. Therefore, introducing hospital resources constitutes a future research direction, which will improve the comprehensiveness of models and generate more precise strategy evaluations.

## 5. Conclusions

In this study, in order to deal with an increasing number of congestion issues, an integrated simulation model for a whole hospital that captures the patterns of patient flows based on real time-series data was used to evaluate decongestion strategies. The outcome indicated that moving ED patients is a preferable method for congestion control from a managerial viewpoint; however, these strategies might lead to patients’ dissatisfaction due to potential longer waiting times. In addition, medical-related units might have an appreciable impact on hospital overcrowding, which requires more attention. Adjusting the size of physical units was more effective for congestion reduction than operating patients. Furthermore, diverting smaller numbers of ambulances (e.g., diverting two arrivals) seems to be more effective and efficient for congestion reduction compared to the combination scenario. If the congestion situation requires the control of further interventions, instead of implementing an isolated approach continuously, combining one approach with other strategies is highly recommended.

## Figures and Tables

**Table 1:** Summary results of scenarios (20 replications).

Scenario No	Mean Occupancy (10 AM)	Mean Std Dev.	Number of Congestion Events	Maximum Congestion Time (h)	Minimum Congestion Time (h)	Mean Congestion Time (h)	Affected Patients	Postponed Elective Patients	Diverted Ambulance Arrivals
0	327.19	4.13	111.75	21.7	2	6.78			
Adding 2, 4, and 6 ED beds									
1	328.57	4.54	85.16	22.42	2	6.89			
2	327.31	4.66	62.65	21.25	2	6.32			
3	325.96	5.22	28.7	17.75	2	6.19			
Adding 2, 4, and 6 AMU beds									
4	328.96	4.12	71.4	21.25	2	6.35			
5	328.93	4.75	46.65	18.45	2	5.5			
6	328.24	4.9	21.35	14.3	2	4.76			
Moving 2, 4, and 6 beds surgical beds to medical units (No change in total numbers of inpatient beds)									
7	327.15	4.56	105.45	22	2	5.71			
8	324.09	4.71	100.15	20.35	2	6.23			
9	322.35	4.55	73.4	20.2	2	6.18			
Moving 2, 4, and 6 beds medical beds to surgical units (No change in total numbers of inpatient beds)									
10	328.66	4.76	107.95	20.95	2	6.25			
11	330.31	4.52	132.53	23.21	2	7.3			
12	330.59	4.39	140.8	22.1	2	6.81			
Moving 2, 4, and 6 ED patients to other hospitals									
13	326.91	4.67	84.15	20.75	2	6.24	299		
14	327.56	4.29	68.65	22.2	2	6.62	422		
15	327.72	4.34	64.2	21.8	2	6.08	438		
Discharging 2, 4, and 6 inpatients									
16	328.12	4.24	100.2	22.3	2	6.3	353.9		
17	327.83	4.22	97.75	21.2	2	6.27	429.7		
18	327.27	4.53	95.2	21.9	2	6.62	452.6		
Postponing 2, 4, and 6 elective surgeries									
19	327.6	4.41	101	18.35	2	5.78		101.1	
20	327.69	4.46	75.5	17.35	2	5.09		112.1	
21	326.87	4.76	70.1	17.8	2	5.06		142.2	
Diverting 2 and 4 ambulances									
22	326.73	4.49	39.55	13.1	2	3.68			316
23	313.85	4.68	29.15	12.5	2	4.5			438.6
Moving 1 and 2 ED patients and Diverting 1 and 2 ambulances									
24	326.32	4.49	51.8	18.45	2	4.71	131.25		161
25	312.85	6.1	31.5	13.8	2	3.31	201.85		342.8
Moving 1 ED patient, diverting 1 ambulance, and discharging 1 surgical and 1 medical patient									
26	314.35	6.12	49.9	17.35	2	4.23	133.15		240.35

**Table 2:** Comparing the efficiency of a single strategy and combined strategies.

Scenario No	Description	Numbers of Affected Patients	The Efficiency in Terms of Reducing Number of Congestion Events	The Efficiency in Terms of Congestion Time Reduction
22	Diverting 2 ambulances	2	0.23	0.0098
23	Diverting 4 ambulances	4	0.14	0.004
24	Moving 1 ED patient and diverting 1 ambulance	2	0.21	0.0071
25	Moving 2 ED patients and diverting 2 ambulances	4	0.15	0.0064
26	Moving 1 ED patient, diverting 1 ambulance, and discharging 2 inpatients	4	0.17	0.0068

## Data Availability

The patient flow data used for this work were obtained with approval by the Ethics Committee, SA Health Office for the Research Study ‘Congestion recovery and optimisation of patient flows’ (Application number 475.13). These data were used under license for the current study; thus, they are not publicly available.

## Supplementary Materials

The following supporting information can be downloaded at: https://www.mdpi.com/article/10.3390/ijerph192316348/s1. Figure S1. Overview of the HESMAD structure; Table S1. The description of physical units of the HESMAD; Table S2. HESMAD process modules; Table S3. Historical data of inpatients.
