# Supplementary material for: Effective Response to Hospital Congestion Scenarios: Simulation-Based Evaluation of Decongestion Interventions
Source: Int J Environ Res Public Health. 2022 Dec 6;19(23):16348. doi: 10.3390/ijerph192316348 (PMC9737001; doi:10.3390/ijerph192316348)
Supplement: Supplementary file 1 [file ijerph-19-16348-s001.zip › ijerph-1947214-supplementary.pdf]

Effective Response to Hospital Congestion Scenarios: Simulation-Based Evaluation of Decongestion Interventions

Wanxin Hou [1,*], Shaowen Qin [2] and Campbell Henry Thompson [3]

[1] School of Information Science and Technology, Research Centre for Intelligent Information Technology, Nantong University, Nantong 226019, China
[2] College of Science and Engineering, Flinders University, Adelaide 5042, Australia
[3] School of Medicine, University of Adelaide, Adelaide 5005, Australia
* Correspondence: houwanxin0403@ntu.edu.cn

Supplementary Information (SI)

SI1. HESMAD structure

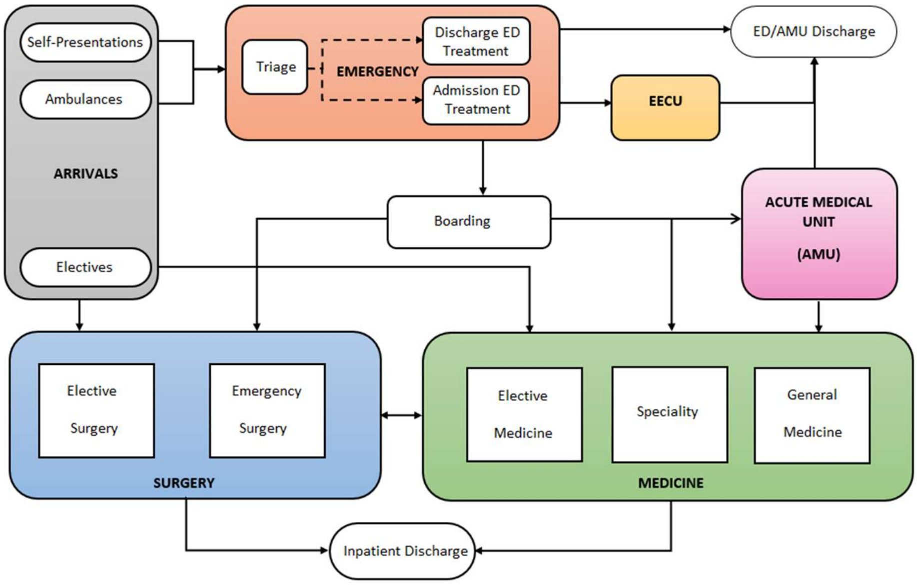

Figure S1 Overview of the HESMAD structure.

SI2.The Process and modules of HESMAD

Different types of patients go through physical units (SI-Table S1) which were represented as modules in the model. All processes, interactions and behaviours between patients and the hospital were included in each module to imitate patient journeys.

The linked modules catch patient pathways when they go through the physical units in the hospital. The process begins when all patients except electives arrive in the ED. A triage score is assigned to indicate the severity of patient condition and to decide treatment stream and queuing priority. Some patients are discharged when they finish ED treatment. Other patients are admitted into the hospital inpatient units. In the inpatient departments, 330 base beds and 8 flexible beds were modeled in the simulation model. Patients are placed in a queue when beds are not available in relevant treatment areas. Probability distributions obtained from historical data were used to reflect patient state and their LOS in different areas of the hospital. The final process of discharging patients involves releasing resources. Monitoring different indicators such as the number of beds occupied allows one to evaluate hospital congestion and investigate the potential influence of strategical interventions on hospital overcrowding.

Table S1 The description of physical units of the HESMAD

**Physical Unit Description**
**1. ED** Emergency department (18 beds for admission stream, 18 beds for discharge stream): models initial care of patients; two arrival modes including self-presentation or by ambulance.
**2. EECU** Extended emergency care unit (10 beds): fast-turnover inpatient ward for patients who require up to 24 hours of monitoring within the ED before being sent home.
**3. AMU** Acute medical unit (30 beds): assessment unit for general medical and acute care elderly patients.
**4.Medicine** Largest inpatient division within the hospital (170 beds). Treats patients requiring emergency hospital care for medical conditions with complex and multi system disease. Three treatment streams are modelled based on how patients enter the division: speciality – referred directly from ED, general medicine – patients underwent AMU assessment and, elective – non-emergency arrival undergoing non-surgical elective procedure requiring overnight care.
**5. Surgical** Other primary inpatient division (130 beds) that treats patients requiring surgery. Two treatment streams based on mode of entry: emergency surgery and elective surgery.

The main state variable we are modelling is occupancy on a given day, $\tau \in N^0$, denoted by vector $\mathbf{X}(\tau)$. Conceptually, the model which computes $\mathbf{X}(\tau, \ell, i)$ reported in Figure S1 is the balance equation.

$$\mathbf{X}(\tau,\ell,\ i) = f(\mathbf{X}(\tau-1,\ell,i),\mathbf{A}(\tau,m),\mathbf{D}(\tau,\ell,\ i)) \quad (1)$$

where $\mathbf{A}(\tau, m)$ and $\mathbf{D}(\tau, \ell, i)$ are vectors representing the arrivals and discharges for day τ, and are realisations of the corresponding stochastic processes that are modelled in their individual process modules. We note that the use of vectors in (1) is indicative of the complex nature of the hospital system where each of the vectors **X**, **A** and **D** comprises sub-populations representing similar events (e.g., arrivals) but vary sufficiently in their physical and modelled behavior. For occupancy **X** and discharge **D**, $\ell \in \{1,\ldots,5\}$ represents a division/area within the hospital where each ℓ corresponds to one of HESMAD's physical units (see SI-Table S1) and $i \in I(\ell) \in N$ represents a particular stream within the division ℓ. For arrivals the vector $\mathbf{A}(\tau, m)$ comprises three elements $Am(\tau)$ where m={1,2,3} represents different mode of patient arrival and corresponds to ambulance, self-presentation and elective arrivals, respectively.

For simplicity, we use the variable $t \in [0, 24)$ to represent the hourly time during day τ, where τ+t actually denotes τ+t/24. When no ambiguity exists, we drop the τ from the argument and simply use the daily time t. Finally, $X\ell(t)=\sum i \in I(\ell)X(t,\ell,i)$ represents the aggregated occupancy across all streams i of physical unit ℓ at time t.

Since HESMAD includes many random variables the issue of the use of parametric versus empirical distributions arises naturally. In constructing and validating HESMAD we used both parametric (e.g., arrival process) and empirical distributions (e.g., see SI-Table S2, triage, ED streams and treatment process modules). There is a natural temptation to use parametric distributions whenever they provide very good fits to observed trends because these may be easily transferrable to analogous variables in other hospitals. We followed standard statistical measures for "goodness of fit" to make the above determination [21].

Table S2 HESMAD process modules

Process Module	Description
**Arrivals**	Generates emergency and elective patients within the simulation.
**Triage**	System for defining the urgency of clinical review and the initiation of definitive treatment. Empirical distributions based on mode of arrival. Patients are assigned a triage upon arrival which ranges from 1-5. A low score indicates requiring immediate attention and a high score corresponds to a less critical case.
**ED buffer**	Post-triage patient queue. Order of service is based on assigned triage score, priority given to triage 1 and 2 patients.
**ED streams**	Assesses, treats and/or stabilizes emergency patients. Contains two, physically separated, streams based on likelihood of admission for inpatient care. Estimated treatment time is drawn from empirical distributions based on ED stream, triage score and mode of arrival.
**Dispatch**	Allocates patients to modelled inpatient streams. Exploits probabilistic distributions influenced by triage score.
**Boarding**	Post ED treatment queue for admitted patients waiting for an inpatient bed. Boarding patients occupy emergency treatment space, reducing ED throughput.
**Treatment**	Simulates a patient's period of treatment associated with their current inpatient stream. LOS is drawn from empirical distributions for relevant stream.
**Resources**	Finite resource utilized by patients within physical units. Resources includes ED treatment spaces and inpatients beds. Restricts patient flow between physical units based on availability.
**Operating hours**	Schedule module which controls inpatient operation hours relevant to various patient streams.
**Discharge**	Controls discharge processes for patients exiting FMC from a physical unit. Includes administrative delay relating to processing a discharge as well as a cleaning delay on freeing up a resource.

SI3. Historical data of inpatients

Table S3 Historical data of inpatients

	Total numbers	Proportion of patients whose LOS<=21 days	Proportion of patients whose LOS>21 days
Medical Patients	21773	94%	6%
Surgical Patients	13630	97%	3%
